# Dealing with Data: A Case Study on Information and Data Management Literacy

**DOI:** 10.1371/journal.pbio.1001339

**Published:** 2012-05-29

**Authors:** Melissa A. Haendel, Nicole A. Vasilevsky, Jacqueline A. Wirz

**Affiliations:** Library, Oregon Health & Science University, Portland, Oregon, United States of America

## Abstract

The launch of the eagle-i Consortium, a collaborative network for sharing information about research resources, such as protocols and reagents, provides a vivid demonstration of the challenges that researchers, libraries and institutions face in making their data available to others.

Our scientific body of knowledge is built upon data, which is carefully collected, analyzed, and presented in scholarly reports. We are now witnessing a dramatic shift in our relationship to data: where researchers once managed discrete, controllable building blocks of knowledge, they must now contend with a tsunami of information that paradoxically feeds the growing scientific output while simultaneously crushing researchers with its weight [1]. Numerous national and international initiatives, projects, and working groups have been established to address the data dilemma from multiple angles [2]–[6], including recent Requests for Information from the US Office of Science and Technology Policy [7] and the National Institutes of Health (NIH) [8], and a US White House announcement of spending US$200 million on “Big Data” [9]. The need for information and data management literacy extends beyond a national mandate for sharing and public access—the scientific community must embrace a culture where every scientist needs to understand how to manage, navigate, and curate huge amounts of data. Libraries have traditionally been the place to acquire information; now they have become the place to learn how to manage it. The eagle-i Consortium (see Box 1), a collaborative resource sharing network, is designed to address both the researcher's data-sharing needs and the modern library's new mandate to facilitate and accelerate the discovery of new knowledge. The launch and development of this initiative provides a vivid demonstration of the challenges that researchers, libraries, and institutions face in making their data available to others.

**Box 1. About eagle-i**

eagle-i is a US$15 million NIH-funded pilot project with the aim of facilitating biomedical research by creating a network of research resources repositories. The Network began with nine institutions chosen on the basis of their diversity and geographical location, and has recently added 16 new institutions (Table 1). The eagle-i platform consists of ontology-driven Semantic Web Entry & Editing Tool (SWEET) [25],[26], which enable resource information contained in Resource Description Framework (RDF) repositories to be published as Linked Open Data [10]. The use of an ontology that integrates domain standards for representation ensures interoperability and semantic linkage of research resources to other aspects of biomedicine. As part of the two-year pilot, each of the original participating institutions employed specialized Resource Navigators at each site to identify relevant research resources and enter data into the system, while a central Biocuration team at the Oregon Health & Science University and Harvard Medical School libraries built the ontologies and ensured the quality and consistency of the data [27]. To date, the eagle-i repositories contain records for over 47,000 resources and additional records are continually added. New institutions are invited to adopt the software and join the network [17]. As eagle-i matures, new strategies are under way to streamline the data collection process, including integration with laboratory inventory systems and with other online resources such as National Center for Biotechnology Information (NCBI) [28] and the Neuroscience Information Framework (NIF) [29].

**Table 1:** Participating institutions in the eagle-i Network.

Original Participating Institutions	Year 3 New Participants
Dartmouth College, Hanover, NH	Charles Drew University, Los Angeles, CA
Harvard University, Cambridge, MA	Clark Atlanta University, Atlanta, GA
Jackson State University, Jackson, MS	Florida Agricultural and Mechanical University, Tallahasse, FL
Montana State University, Bozeman, MT	Howard University, Washington, DC
Morehouse School of Medicine, Atlanta, GA	Hunter College, New York, NY
Oregon Health & Science University, Portland, OR	Meharry Medical College, Nashville, TN
University of Alaska Fairbanks, Fairbanks, AK	Ponce School of Medicine, Ponce, PR
University of Hawaii at Manoa, Manoa, HI	Texas Southern University, Houston, TX
University of Puerto Rico, San Juan, PR	The City University of New York, New York, NY
	The University of Texas at El Paso, El Paso, TX
	The University of Texas at San Antonio, San Antonio, TX
	Tuskegee University, Tuskegee, AL
	Universidad Central del Caribe, Bayomon, PR
	University of Pennsylvania, Philadelphia, PA
	Vanderbilt University, Nashville, TN
	Xavier University, New Orleans, LA

## Scholarly Communication

The scholarly communication cycle refers to the process where scholars create, share, and preserve their research. The nature of this cycle has changed dramatically over the past decade. For example, the NIH public access policy has dramatically altered the relationship between researchers and publishers by mandating public access to all peer-reviewed publications of NIH-funded research. Similarly, many government agencies now require a data-sharing plan as part of an application for funding. In an era of Linked Open Data and the Semantic Web [10], research today comprises information in many forms: blogs, tweets, database entries, and grant reports that could be made available as Linked Data. The launch of new initiatives to accelerate publication and use of new and emerging technologies to enable improved data presentation [11] has spurred further conversations about enabling data-driven “publications” whereby the data itself is cited [12]. Further, it has been suggested that publications should be evaluated based on whether they have enriched content to provide interactivity, available datasets, and machine-readable metadata [13].

As the types and variety of data have changed, so too has the role of data in scholarly communication. New and emerging issues surrounding the volume, storage, sharing, and cataloging of data have created major bottlenecks in the scholarly communication cycle [14]. The enormity of data available to scientists provides incredible opportunities for innovative research, but maintaining and navigating such datasets poses major obstacles. A recent survey reported that 85% of scientists surveyed are interested in using other researchers' data, but only 36% report their own data is easily accessible [15]. Scientists today need to rely on data management not just at the end of a project, but during its whole life cycle. Thus, it's imperative that we develop the tools to handle data effectively and efficiently as we continue to consume and generate it. As a step towards facilitating quality data management practices, NIH has recently announced support for informationists to work on currently funded research grants [16].

## From Plan to Practice: The eagle-i Network

The eagle-i Network aims to accelerate the cycle of scholarly communication by making research resources easy to find—including resources that are generated in the course of research and sit on the lab bench, on shelves, or in freezers. Toward that end, “Resource Navigators” at participating institutions (Box 1) gathered information from individual laboratories regarding protocols, organisms, reagents, instruments, services, human studies, software, research opportunities, and biospecimens. Information about these resources was then made available publicly through a semantically enabled search application [17].

Resource sharing of this nature requires a level of documentation and organization that, in our experience, research laboratories rarely implement. Consequently, even though we found researchers were largely willing to share resources, it took considerable effort to first gather and structure the data. If we could ensure that resources are consistently tracked during the course of research and data generation, it would make it much easier to disseminate information about resources via publications, grant reports, database entries, etc. Uniquely identifying research resources is critical both to enable sharing and to ensure reproducibility of science. Currently, there is no standard method for keeping track of data and resources within academic labs. Some labs use formal laboratory inventory management systems (LIMS), such as Accelerated Technology Laboratories, Inc. or LABLynx, but these tend to be too expensive and cumbersome for most academic use. In fact, we found that 85% of the labs visited at Oregon Health & Science University as part of the eagle-i project did not indicate use of a lab inventory system. Furthermore, labs that do track resources typically use an informal, often distributed system of spreadsheets or applications such as Microsoft Access or Filemaker Pro. These informal tracking systems often do not contain detailed enough information about a lab's resources that would allow for unique identification and semantic linking to other data, such as the source organism or GenBank accession number for a plasmid insert. Most researchers could not provide this information, as it was not readily on hand or often unknown.

To make semantically structured data available without exhaustive external work by specialized staff, we recommend that existing and future resource information be recorded and organized in the context of the laboratory. This will ensure that the resources can be imported into other systems, such as eagle-i, and be permanently associated with any resulting publication—a critical aspect of scientific reproducibility. Many existing websites and repositories such as the National Center for Biotechnology Information (NCBI) Taxonomy for organisms or EntrezGene for genes, for example, use controlled vocabularies and unique identifiers that would support easy import. Unfortunately, we do not yet have the scientific culture to incorporate such metadata into one's research, even at publication time, and thus ensure such interoperability. Journals, reviewers, and funding agencies require little if any reference to semantic entities and researchers are largely familiar with them only in the context of searching databases.

## Creating a Culture of Semantic Scientists

Researchers often don't realize that their own scholarly communications constitute a primary source of data available in public databases. Because researchers know their data best, one solution is for them to tag their own data using universally agreed upon standards [18]. One effort to address this issue was a 2011 workshop called “Beyond the PDF” [19], aimed at identifying what requirements scholars would need to mark up their published works. This would reduce the burden of information management and interpretation by the army of biocurators currently required to deal with the output of scholarly communication. A complementary approach is to enable researchers to identify the components of their research *during* the course of research by using laboratory project management software such as Labguru, Quartzy, and Syapse. These applications allow principal investigators to manage different projects, resources, data, inventorying, scheduling, etc. amongst different members of the lab. If these systems became more commonplace and were able to record uniquely identified entities and link data between different resources, this information could be published as Linked Open Data and used both as immediate reference for scholarly communication as well as to feed resource discovery systems such as eagle-i.

Despite a commonly acknowledged need for database and data management solutions, the lack of community buy-in remains the main obstacle to any large-scale implementation of bioresource curation and development. As the eagle-i experience has shown, a national top-down mandate for data sharing has not generated data management plans at a laboratory level. One reason for this seems to be a lack of clear incentive. As research scientists, we take particular pride in our intellectual autonomy and our mastery of the techniques necessary to answer our scientific queries. To provide scholars with the incentive to share information and data, therefore, we must focus on purpose: biocuration skills need to be developed not as a quick Band-Aid to retrospectively address national mandates, but rather, because it will generate new insight and advance scientific discovery. Moreover, just acquiring the skill to navigate the complex landscape of different data will become a motivating force in itself.

Projects such as the Bioresource Research Impact Factor (BRIF) have been proposed to recognize scientific contributions to the development and maintenance of bioresources, as well as to quantitatively track the use and impact of specific resources [20]. Related is the notion of a “nanopublication,” wherein tables, graphs, and other data are represented with their own unique identifiers and are linked with provenance to their source [21],[22]. Such “data journals” hold promise for motivating scientists because it formally recognizes their ability to provide structured data. Recognition may well prove to be a more sophisticated and ultimately more successful method than federal mandates, and could bring the scientific community to a new level of information literacy. However, we argue that early education in statistics, ethics, and data and information literacy should accompany scientific training to establish a new cultural standard.

Beyond such management issues, the explosive growth of data has also introduced new ethical considerations. As we establish new methods of managing data, it is equally important to develop standards of ownership and development that clarify the roles and responsibilities of researchers. Dr. Palmer of UC Irvine has observed that “Currently, if you use a library's Special Collections department, you get white glove services to find, use, understand, and appreciate the provenance of the resource. But if you want to use data, there's no one to help you.” However, Dr. Palmer believes that data will soon be like other library collections, which have evolved standards and ethical guidelines. Numerous libraries are now working to support their local research communities better with respect to data access and discovery. Spending money and time on data management, valuing the scientists that perform this work, and using science to prove the value of organized and shared data are all required to change this attitude [23]. This will not only foster a responsible approach to personal data management but will also facilitate collaboration between scientists and scientific reproducibility, as data sharing becomes less onerous and more productive. Funding agencies must also recognize the need to support adequate information management when making funding decisions and providing guidance in everything from training programs to research grants.

Libraries are an under-recognized resource in the field of data and information literacy. Librarians have increasingly become experts in data management because of their combined knowledge of new data sharing standards, information science, and the Semantic Web [24]. For instance, the eagle-i curation team consists of Semantic Web experts, ontologists, librarians, and domain curators. Information literacy has always been a topic of interest to research librarians, and it is natural that their role is expanding to include topics surrounding data curation and access. The sustainability of any long-term bioresource curation project requires an institutional level of support that permeates new standards of information and data literacy into the local culture; the library can serve as an important nexus to help educate and promote data and information literacy at the university. Librarians not only educate the community on data and information literacy, but conduct their own research on how the scientific community can best rise to the data challenge. As scientists continue to adapt to the ever-changing data landscape, it is important that we develop and share appropriate tools and techniques to organize and access the information that is the foundation of our scientific endeavors. The solution may be as close as your local library.

## Abbreviations

BRIF: Bioresource Research Impact Factor
LIMS: Laboratory Inventory Management Systems
NCBI: National Center for Biotechnology Information
NIF: Neuroscience Information Framework
NIH: National Institutes of Health
RCMI: Research Centers in Minority Institutions
